# ACADS acts as a potential methylation biomarker associated with the proliferation and metastasis of hepatocellular carcinomas

**DOI:** 10.18632/aging.102292

**Published:** 2019-10-25

**Authors:** Diyu Chen, Xiaode Feng, Zhen Lv, Xiaofeng Xu, Yuejie Lu, Wenxuan Wu, Hao Wu, Hua Liu, Linping Cao, Sunyi Ye, Jianzhong Chen, Jian Wu

**Affiliations:** 1 Division of Hepatobiliary and Pancreatic Surgery, Department of Surgery, First Affiliated Hospital, School of Medicine, Zhejiang University, Hangzhou 310003, Zhejiang, China; 2 Institute of Immunology School of Medicine, Zhejiang University, Hangzhou 310058, Zhejiang, China; 3 Department of Urology, The First Affiliated Hospital, School of Medicine, Zhejiang University, Hangzhou 310058, Zhejiang, China

**Keywords:** HCC, methylation, proliferation, metastasis, DNMT

## Abstract

Background: Hepatocellular carcinomas (HCC) constantly rank among the malignancies with the highest death tolls on the global scale. Moreover, HCC are associated with a limited set of therapeutic options. This is particularly true in the case of advanced stage cancers, where long-term survival is uncommon. For the inoperable, advanced HCC patients, chemotherapy is the main modality of treatment. Due to the lack of known molecular targets, the efficacy of the chemotherapy is limited.

Conclusion: These findings clearly indicate that DNA methylation plays a key role in regulating ACADS expression and that it can be a potential therapeutic target for treating HCC.

Materials and methods: A thorough comparative analysis of 282 cancer samples with 47 normal samples from GEO datasets resulted in the observation that that the level of ACADS was significantly downregulated in HCC. Loss-of-function analyses were then conducted to understand the biological function of ACADS in HCC. It was noted that ACADS was involved in the proliferation and metastasis of HCC. Experiments involving the knockdown of DMNT expression led to the discovery that the expression of ACADS in the HCC cells was significantly increased. The TCGA database was then employed to identify tumor tissue samples which showed higher methylation levels at cg01535453, cg08618068, and cg10174836 (which are the target sites of the ACADS CpG islands) as compared with normal liver tissue samples. All these findings indicated that ACADS might be a novel methylation biomarker associated with HCC.

## INTRODUCTION

Hepatocellular carcinoma (HCC) is the primary form of malignant liver cancer and it is a leading cause of cancer-related deaths globally [1]. HCC has become the seventh most common type of cancer worldwide. About 90% of HCC arise from chronic inflammation that is associated with continuous hepatic injury and hepatocyte regeneration [2, 3]. Surgery is the best course of treatment for HCC patients, but when we are faced with patients suffering from advanced stage HCC, the available therapeutic options are limited [4]. Therefore, understanding the molecular pathogenesis of hepatocellular carcinoma is quintessential for the treatment of HCC [3, 4].

The fact that the etiology and pathogenesis of hepatocellular carcinoma still remain unknown is indeed a significant obstacle. In recent years, hepatocellular carcinoma tumorigenesis and its subsequent development have been strongly associated with genetic mutations [5, 6], epigenetic modifications and alterations to key signaling pathways [7]. Epigenetic modifications, along with genetic alterations, have long been suspected as the most important mechanisms associated with carcinogenesis [7, 8]. Many studies have revealed that the epigenetic modifications, especially variations in the methylation patterns might be the key element in the oncogenic transformation of HCC [9, 10]. Hence, it is of great significance to identify potential methylation biomarkers to assist us with the diagnosis and prognosis of HCC.

The enzymes coded by the genes of the acyl-CoA dehydrogenase (ACAD) gene family are distinguished by the metabolic pathways in which they participate, and by their substrate specificity. The ACAD enzymes constitute a large, pan-taxonomic protein family whose genes are located on the long arm of chromosome 12 (12q22). The gene consists of 10 exons and 1,236 nucleotides [11, 12]. But, the most commonly reported members of this family are not assigned to a specific subfamily, and very little is known about the taxonomic distribution and the evolution of the subfamilies [12]. Five subfamilies of the ACAD gene family participate in the β-oxidation of fatty acids and they exhibit optimal enzymatic activity based on their acyl-CoA substrates of different chain lengths. Based on their preferred acyl-COA substrates, the enzymes of the ACAD family can be classified into, short (ACADS), medium (ACADM), long (ACADL), or very long (ACADV and ACADV2) [13, 14] variants. Upon conducting further studies on tumor pathogenesis, it was noted that the metabolic reactions which occur might play an important role in carcinogenesis. Being one of the key metabolic enzymes associated with the metabolic reactions involved in carcinogenesis, it is highly likely that ACADS could be a novel therapeutic target and therefore it is important to clearly identify the role of ACAD enzymes in carcinogenesis. As of now, the role of ACADS in the progression of HCC is still unclear.

The current study is focused on discovering potential DNA methylation biomarkers associated with HCC and thereby provide novel therapeutic targets for HCC. This was accomplished by systematically utilizing different bioinformatics techniques, which led to the discovery that ACADS could act as a potential diagnostic and prognostic biomarker for HCC. Loss-of-function analysis was then employed to reveal the biological function(s) of ACADS in HCC cells and also to validate our findings. The loss-of-function analysis confirmed that the silencing of ACAD genes in HCC cells upregulated the processes of cell proliferation, migration, and invasion in vitro. Furthermore, it was noted that tumor growth was modulated in vivo, when ACADS was silenced. In addition, it was also observed that the promoter methylation level of ACADS could be regulated by DNA methyltransferases 1/3A/3B (DNMT1/3A/3B) in HCC. Our results demonstrated that ACADS acts as a potential methylation biomarker and promotes the proliferation and metastasis of HCC.

## RESULTS

### ACADS might be a potential biomarker for the diagnosis and prognosis of HCC

**Figure 1:**
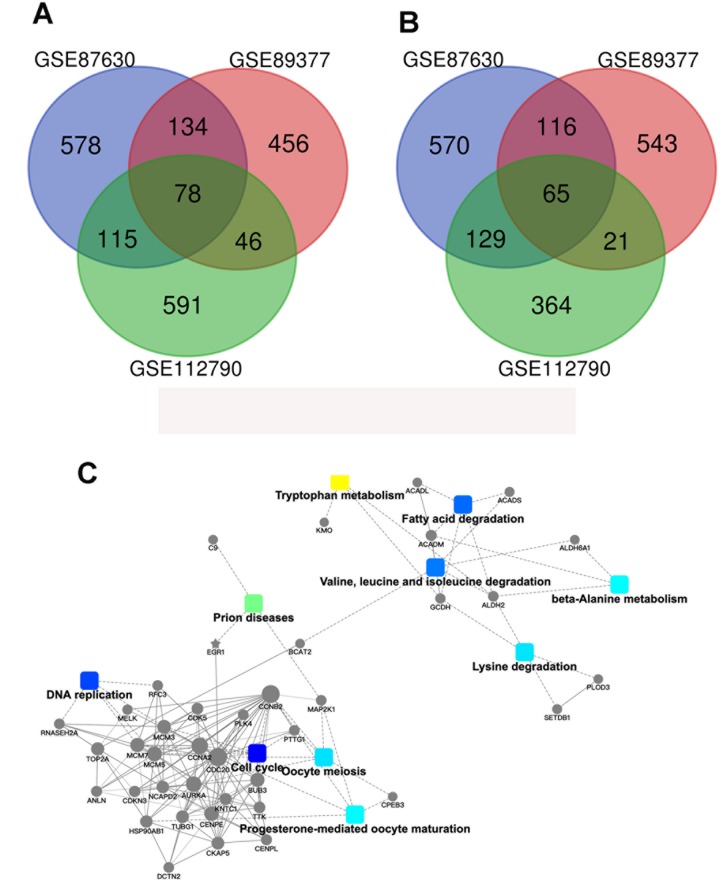
**Identification of the genes which show differential expression patterns in HCC.** (**A**) A Venn diagram of the common DEGs associated with GSE87630, GSE89377, and GSE112790. (**B**) Protein–protein interaction networks which are associated with the differentially expressed genes.

**Figure 2:**
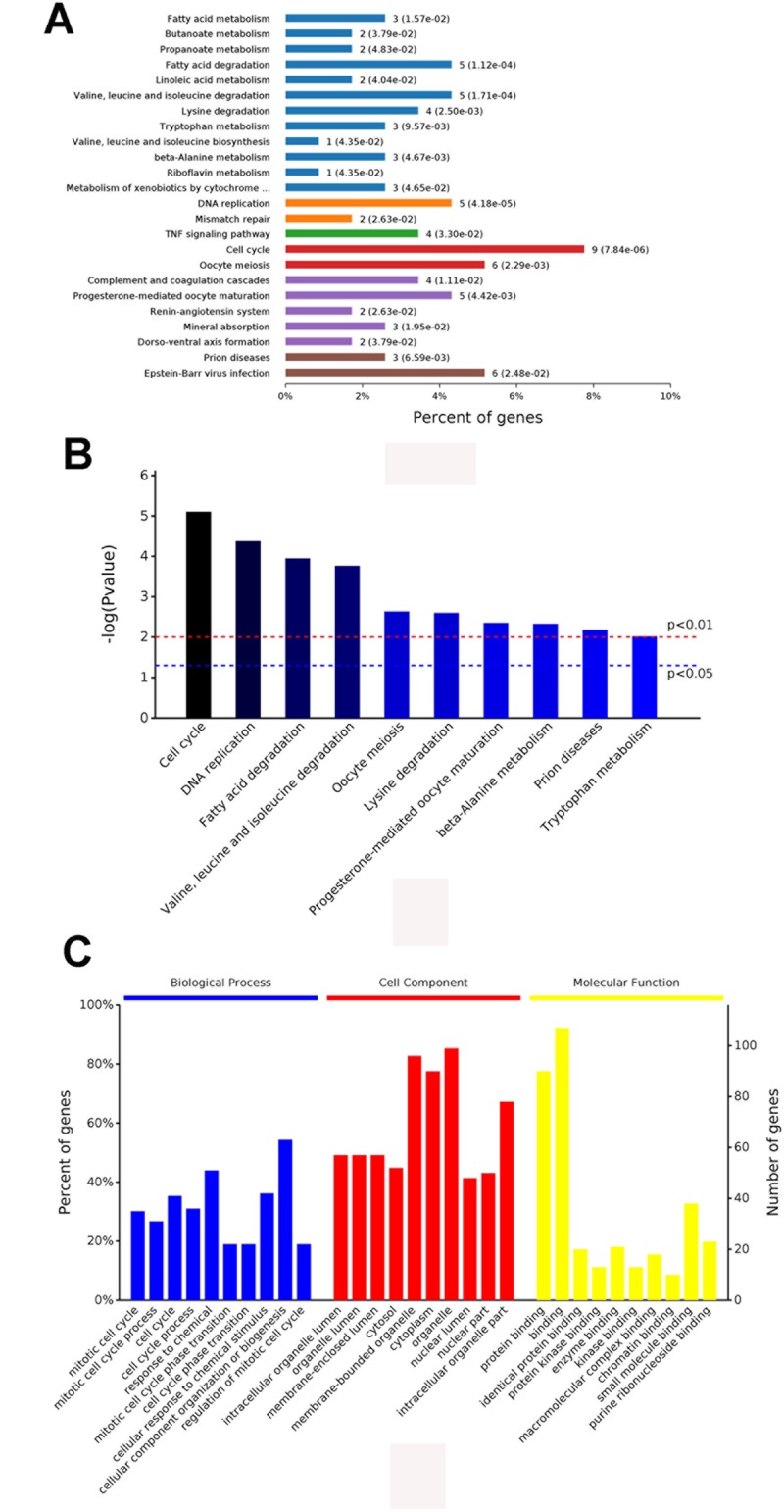
The results of the GO (**A**) and the KEGG pathway enrichment analyses (**B** and **C**) for the identification of DEGs in HCC.

329 samples and their gene expression profiles were obtained from the GEO database (GSE112790, GSE89377, GSE87630). Out of these 329 tissue samples, it was noted that 282 samples were HCC samples whereas the rest i.e., the other 47 tissue samples consisted of normal tissues. It was noted that 78 genes were significantly upregulated and 65 genes were significantly downregulated in the HCC samples (|logFC|≥2, *P*<0.05, Figure 1A). Further screening was performed and the samples were screened based on the level of methylation, i.e., the methylation levels were used as an important biomarker and GO function analyses and KEGG pathway enrichment analyses were performed with the help of DAVID (whose characteristics have been mentioned earlier in the materials and methods section). As shown in Figure 2A and 2B, the KEGG analysis results showed that the downregulated DEGs were found to be mainly associated with the cell cycle and with other cellular processes such as oocyte miosis, DNA replication, fatty acid degradation, valine, leucine and isoleucine degradation process. Moreover, downregulated DEGs were also found to be associated with Epstein-Barr virus infection. (Figure 2C).

In order to discover the genes and the proteins associated with the epigenetic modifications which occur in HCC (these may serve as potential targets for therapeutic intervention), protein-protein interaction (PPI) network analyses were performed (Figure 1B). The results of these analyses were then visualized with the assistance of Cytoscape (http:/www.cytoscape.org/). After evaluating the interactions and the connections between the downregulated DEGs, the top 10 core candidate genes were identified (Table 1). It has to be mentioned that ACADS ranked first in the PPI interaction connectivity score ranking. Besides this, the GEPIA database was utilized to validate the expression patterns of these 10 “hub genes”. The results indicated that the expression of ACADS, KMO, CYP2E1, ACSM3, and EGR1 was significantly higher in the normal tissues as compared to the expression levels seen in HCC tissues (Supplementary Figure 1A). In order to determine the clinical relevance of the key downregulated genes in the HCC samples, the Kaplan-Meier survival analysis was performed and it was observed that low levels of ACADS, ALDH2, and CYP2E1 were associated with poor prognosis among the patients with HCC (Supplementary Figure 1B). Besides, through using cell line webtool, we checked the ACADS expression in 2 normal liver cells and 7 HCC cell lines. Compared with normal liver cells, HCC cells showed lower ACADS expression (Supplementary Figure 2A). Together, all these results highlight the clinical significance of ACADS in HCC, and support the idea that ACADS could be a useful potential biomarker for HCC diagnosis and prognosis.

**Table 1:** Top ten hub genes with higher degree of connectivity.

**Gene symbol**	**Gene description**	**Degree**
ACADS	Acyl-CoA dehydrogenase short chain	18
ACADM	Acyl-CoA dehydrogenase medium chain	17
KMO	Kynurenine 3-monooxygenase	16
ALDH2	Aldehyde dehydrogenase 2 family member	10
ESR1	Estrogen receptor 1	8
MAP2K1	Mitogen-activated protein kinase kinase 1	8
GCDH	Glutaryl-CoA dehydrogenase	7
CYP2E1	Cytochrome P450 family 2 subfamily E member 1	6
ACSM3	Acyl-CoA synthetase medium chain family member 3	5
EGR1	Early growth response 1	5

### ACADS suppress HCC cell proliferation, migration and invasion *in vitro*

**Figure 3:**
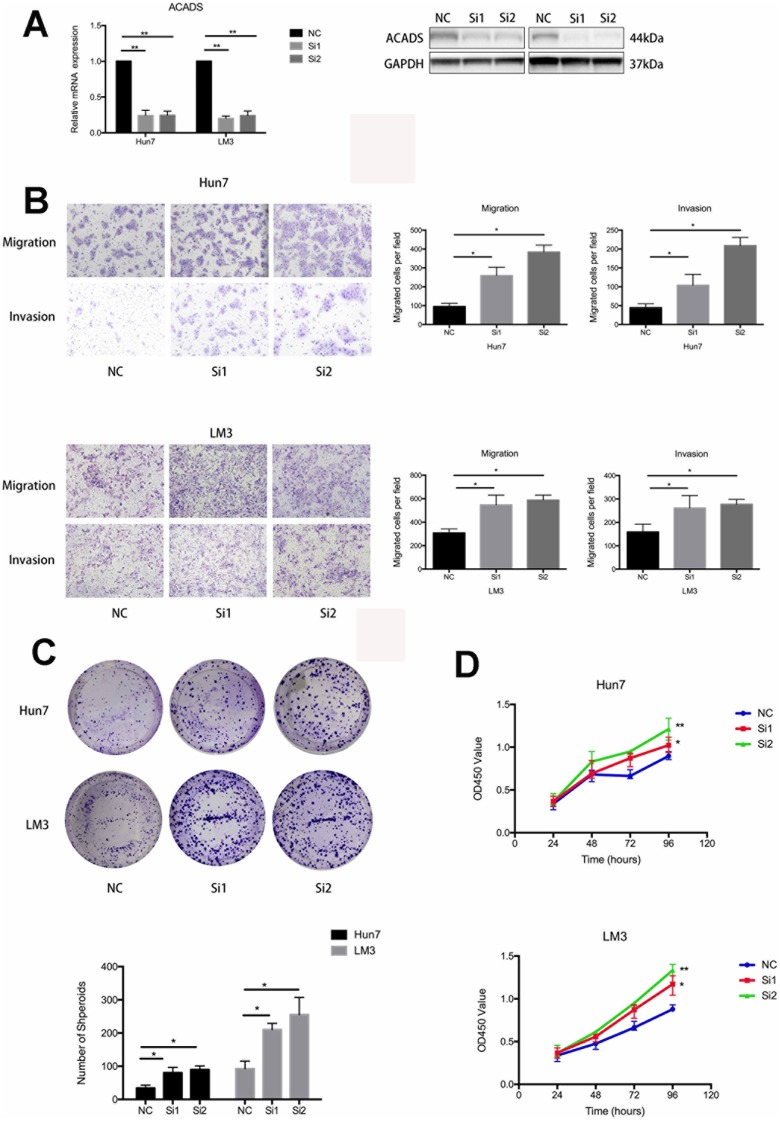
**ACADS is associated with the proliferation, migration, and invasion of HCC cells.** (**A**) The corresponding figure shows that siRNAs downregulate the mRNA and protein expression of ACADS in Huh7 and HCCLM3 cell lines. (**B**) The knockdown of ACADS significantly enhances both the migration and invasion potentials in Huh7 and HCCLM3 cells. Representative images are shown at the bottom. (**C** and **D**) CCK-8 assays and colony assays showed that the proliferation of HCC cells was enhanced following the knockdown of ACADS. (* *P*<0.05, **P<0.01).

Two siRNAs which specifically target ACADS were designed to validate the findings of this study. As shown in Figure 3A, ACADS was effectively knocked down by the siRNAs in the Huh7 and HCCLM3 HCC cell lines and this can be confirmed by checking the mRNA and protein expression levels associated with ACADS. With the help of the transwell assay experiments, it was noted that silencing the ACADS gene increased the migration ability of the HCC cell lines. The results of matrigel invasion assay indicated that knocking down ACADS also facilitated the invasion of HCC cells (Figure 3B). To further analyze the effect of ACADS on cell proliferation, a cell CCK8 assay and a colony formation assay were employed to explore the effect of silencing ACADS on the proliferative ability of Huh7 and HCCLM3 cells. The results indicated that silencing the ACADS gene significantly reduced cell proliferation and colony formation in HCC cells in vitro (Figure 3C and 3D). In addition, we also performed the gain of function assays to ensure the role of ACADS in HCC. As shown in Supplementary Figure 3, followed by the overexpression of ACADS, the ability of proliferation, migration and invasion were reduced in Huh7 and HCCLM3 cells. Therefore, this confirms that ACADS plays the role of a tumor suppressor gene and is involved in suppressing the proliferation, migration and invasion of HCC cells *in vitro*.

### ACADS promotes the apoptosis of HCC and inhibits tumorigenesis under in vivo conditions

**Figure 4:**
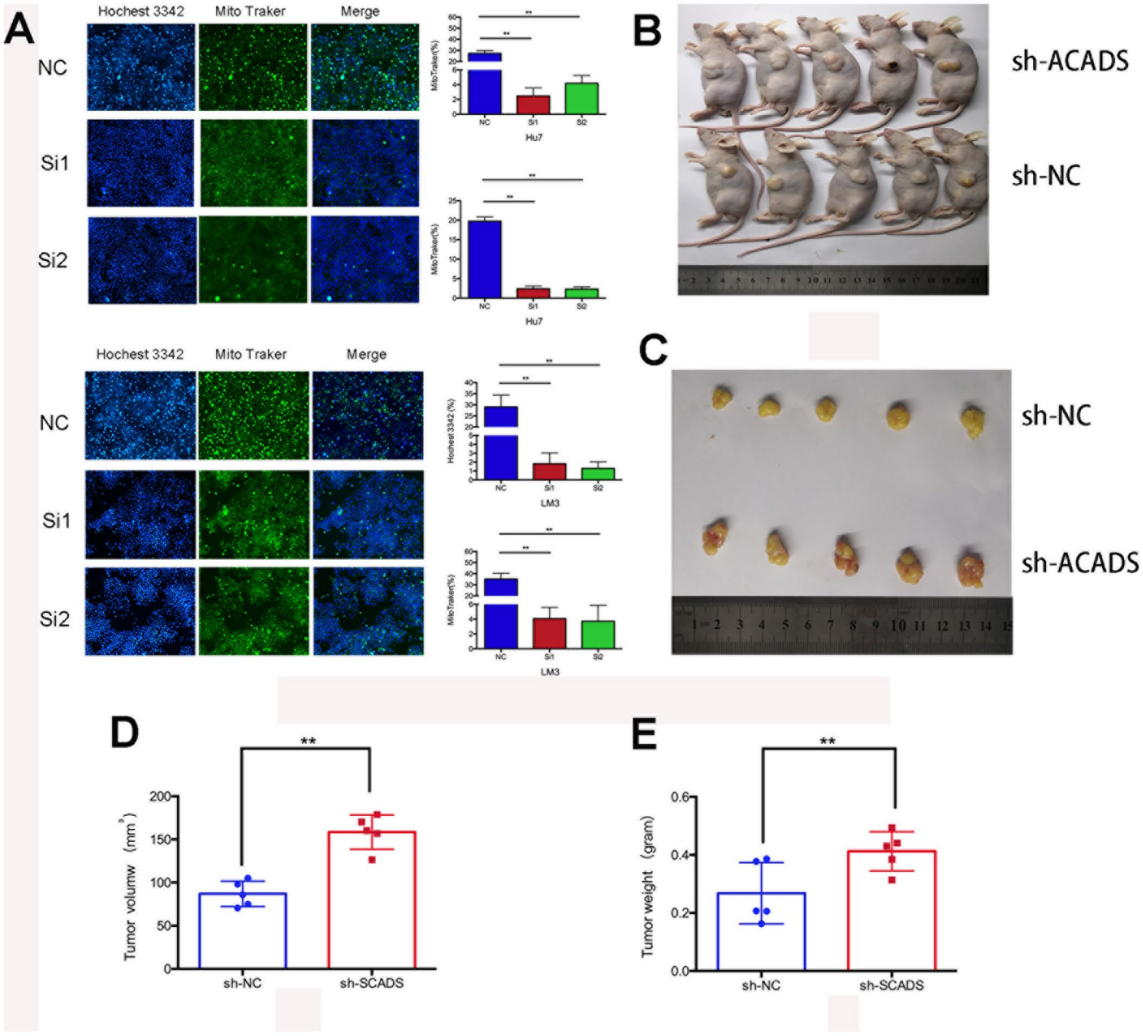
**ACADS promotes apoptosis and it suppresses tumorigenicity in HCC.** (**A**) With the help of the Hochest 3342 and the Mito Traker staining assays, it can be observed that apoptosis was reduced by the knockdown of ACADS in Huh7 and HCCLM3 cells. (**B** and **C**) The Tumor xenograft model. When Tumor xenografts were designed and used, it was noted that the tumors in the shACADS group showed significantly faster growth rates as compared to those tumors which belonged to the shNC group. The typical tumor section samples were isolated and analyzed. (**D** and **E**) A Statistical analysis was conducted which indicated that there was a significant reduction in tumor size and weight. (* *P*<0.05, **P<0.01).

Apoptosis is one of the main factors which affects the proliferation of cancer cells. Nuclear condensation, the lack of inflammation and DNA fragmentation are the hallmarks of cell apoptosis. In this study, the potential role of ACADS in apoptosis was studied after transfecting Huh7 and HCCLM3 cells with ACADS siRNA or control siRNA. A mitotracker and hochest3342 combined staining assay was then used to confirm the apoptotic changes in the two cell lines after transfection. After transfecting the cells with the siRNAs, the cells were incubated in a serum - free medium for 24 hours. The ACADS silenced HCC cells presented lower rate of apoptosis than their control group counterparts (Mitotracker: the siACADS group showed weaker green fluorescence than NC group; Hochest3342: the siACADS group showed weaker blue fluorescence than NC group; Figure 4A). Next, experiments were conducted to check whether ACADS contributes to tumorigenesis in HCC. To assess the functional consequences of ACADS knockdown on tumorigenesis in HCC cells *in vivo*, the tumor initiation capacity of HCC cells transfected with ACADS shRNA, or negative control shRNA (shNC) were examined and compared. Over the course of 35 days, the results of the tumor volume and weight analysis revealed that the ACADS-silenced HCCLM3 cells generated larger subcutaneous xenograft tumors in nude mice as compared to the cells transfected with control shRNA (*P*<0.05; Figure 4B–4D). The results demonstrated that ACADS could suppress the tumorigenicity of HCC *in vivo*.

### Downregulation of ACADS in HCC cells is associated with promoter hyper-methylation

**Figure 5:**
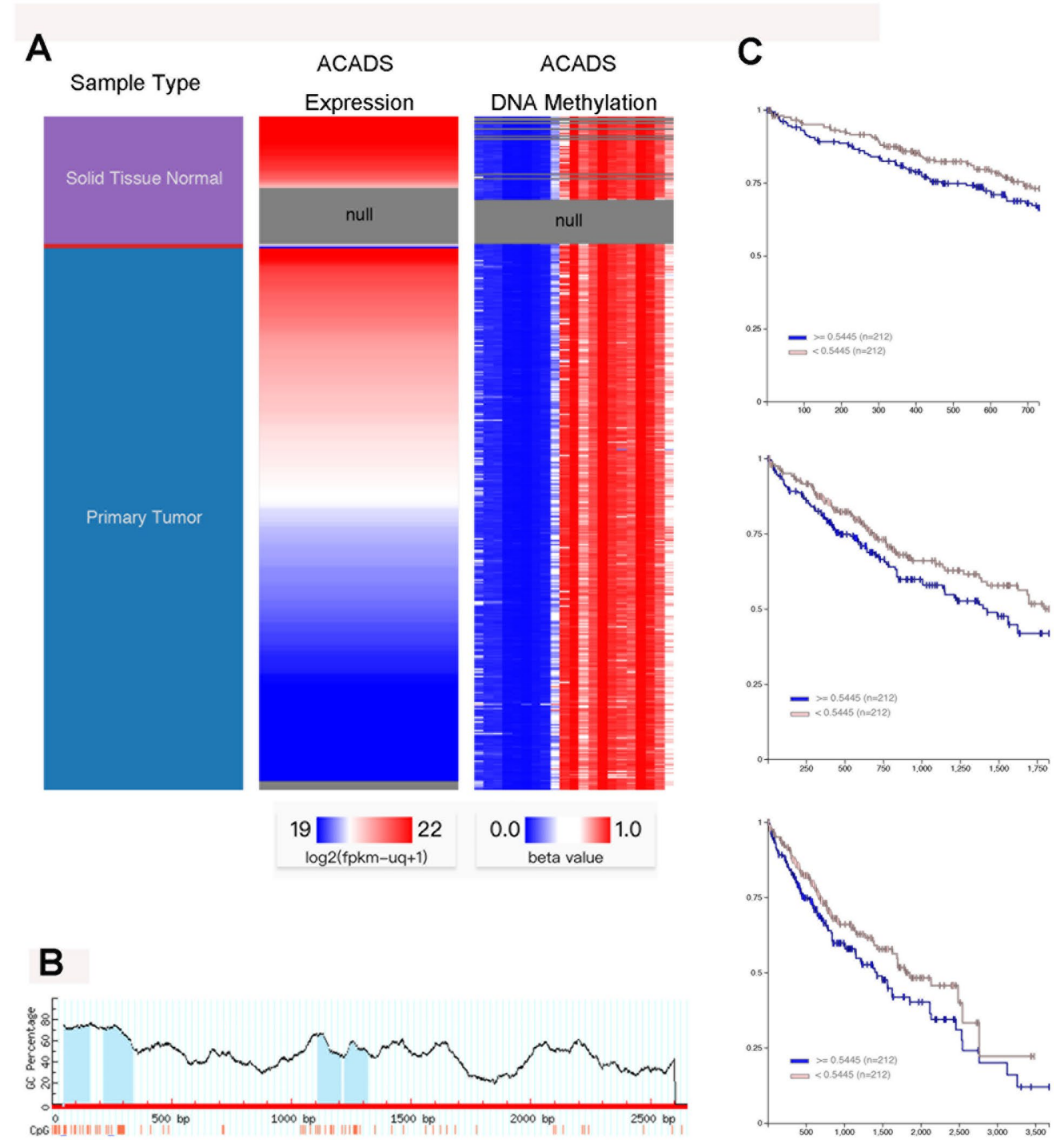
**The downregulation of ACADS in HCC cells is associated with promoter hyper-methylation.** (**A**) From the data obtained with the help of the UCSC Xena database, it can be seen that the normal liver tissues showed higher ACADS expression as compared to their HCC counterparts. It can also be noted that the DNA methylation levels were negatively correlated with ACADS expression in HCC tissues. (**B**) This figure shows the CpG islands of ACADS in the promoter sequence. (**C**) This figure shows that high DNA methylation levels of ACADS are associated with poor 2-, 5- and 10-year survival rates in HCC patients. (* *P*<0.05, **P<0.01).

DNA methylation is a gene silencing mechanism which is implicated in various biological processes, and the CpG islands in gene promoter regions are prone to methylation for a wide variety of reasons [15, 16]. In this study, the relationship between the ACADS and DNA methylation was investigated. By utilizing the of the UCSC Xena database, it was discovered that normal liver tissues showed higher ACADS expressions than HCC tissues, and the DNA methylation level was negatively correlated with the ACADS expression in HCC (Figure 5A). Next, Meth Primer was used to study and identify methylation sites in the ACADS CpG island. It was observed that there were four methylation sites in the ACADS CpG island (Figure 5B). Furthermore, in order to evaluate the relationship between the ACADS DNA methylation level and its prognostic value, HCC DNA methylation sequences from TCGA were used. Figure 5C depicts our findings which note that as compared to the patients with low levels of ACADS DNA methylation, the patients with high DNA methylation levels of the ACADS gene were associated with a worse 2-, 5-, and 10-year survival rate among the patients with HCC. And based on the methylation level of ACADS, we established the nomogram of HCC using TCGA database (Supplementary Figure 2B). We found that the methylation of ACADS impacted the 2- and 5-year survival rate of HCC patients. All the results obtained from the experiments pertinent to this topic conducted during the course of this study indicated that the downregulation of ACADS in HCC cells is associated with promoter hyper-methylation. And the ACADS expression and its methylation level may have the prognostic value of HCC patients.

To further understand the mechanism of CpG island methylation of ACADS in HCC, HCC MEDIP sequence data was collected from TCGA and analyzed (the samples used for extracting this data included 204 HCC samples and 35 normal liver tissue samples). The results and observations of these analyses are shown in Supplementary Figure 4, and from the results it can be noted that the tumor tissues showed higher methylation levels when compared with the normal liver tissues in the ACADS CpG islands - cg01535453, cg08618068, and cg10174836 and therefore these three sites are the target sites of the ACADS CpG island. All these results indicate that the downregulation of ACADS in HCC cells is associated with promoter hyper-methylation.

### The CpG island methylation of ACADS is regulated by DNMTs in HCC

**Figure 6:**
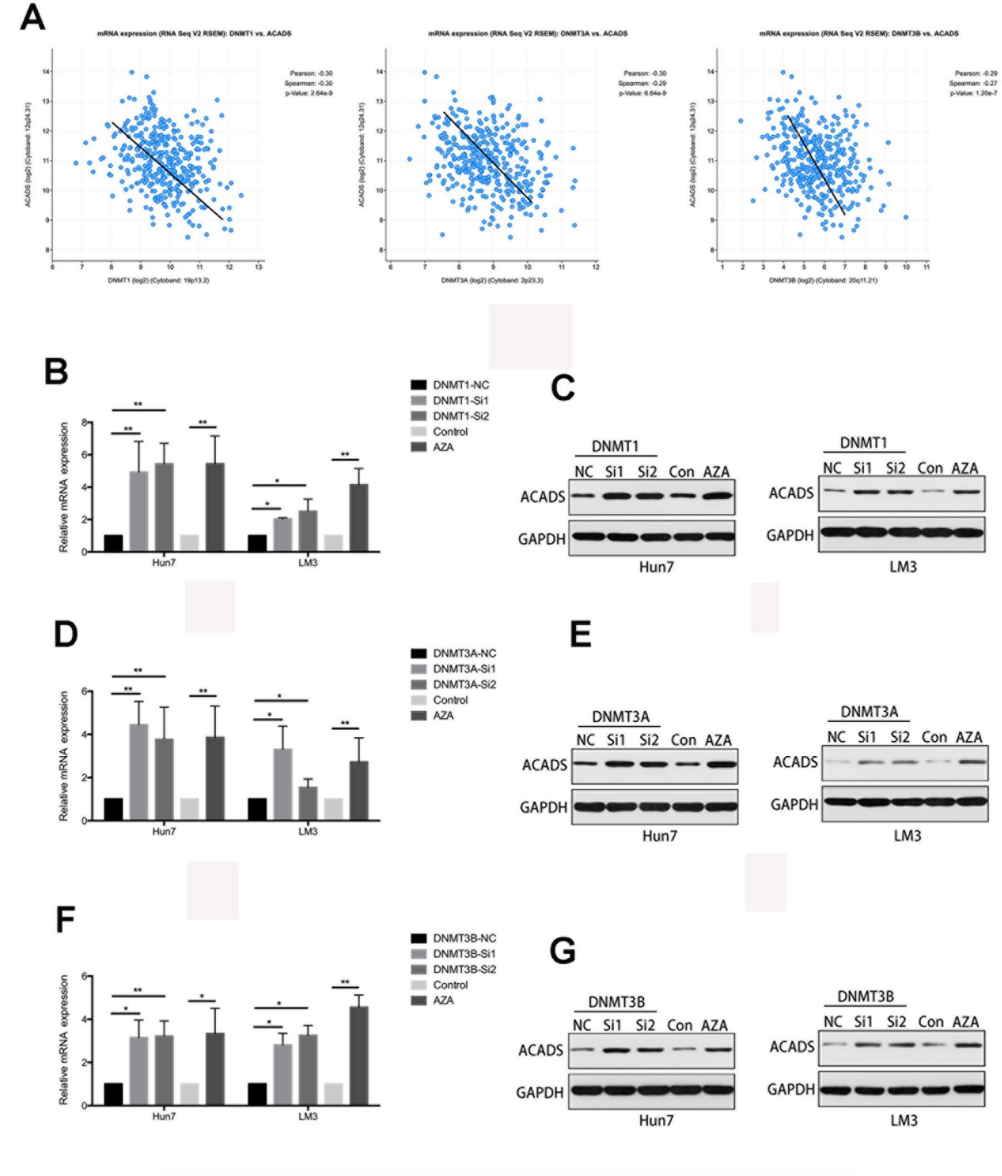
**The CpG Island methylation levels of ACADS are regulated by DNMTs in HCC cells.** (**A**) According to the results obtained with the help of the Starbase database, the expression of ACADS is negatively correlated with that of DNMT1, DNMT3A, and DNMT3B. (**B–G**) The expression of ACADS in HCCLM3 and Huh7 cells was significantly increased after treatment with 5-Aza. It can also be seen that the mRNA and protein expression of ACADS were both increased if the DMNT genes were silenced. (* *P*<0.05, **P<0.01).

**Figure 7:**
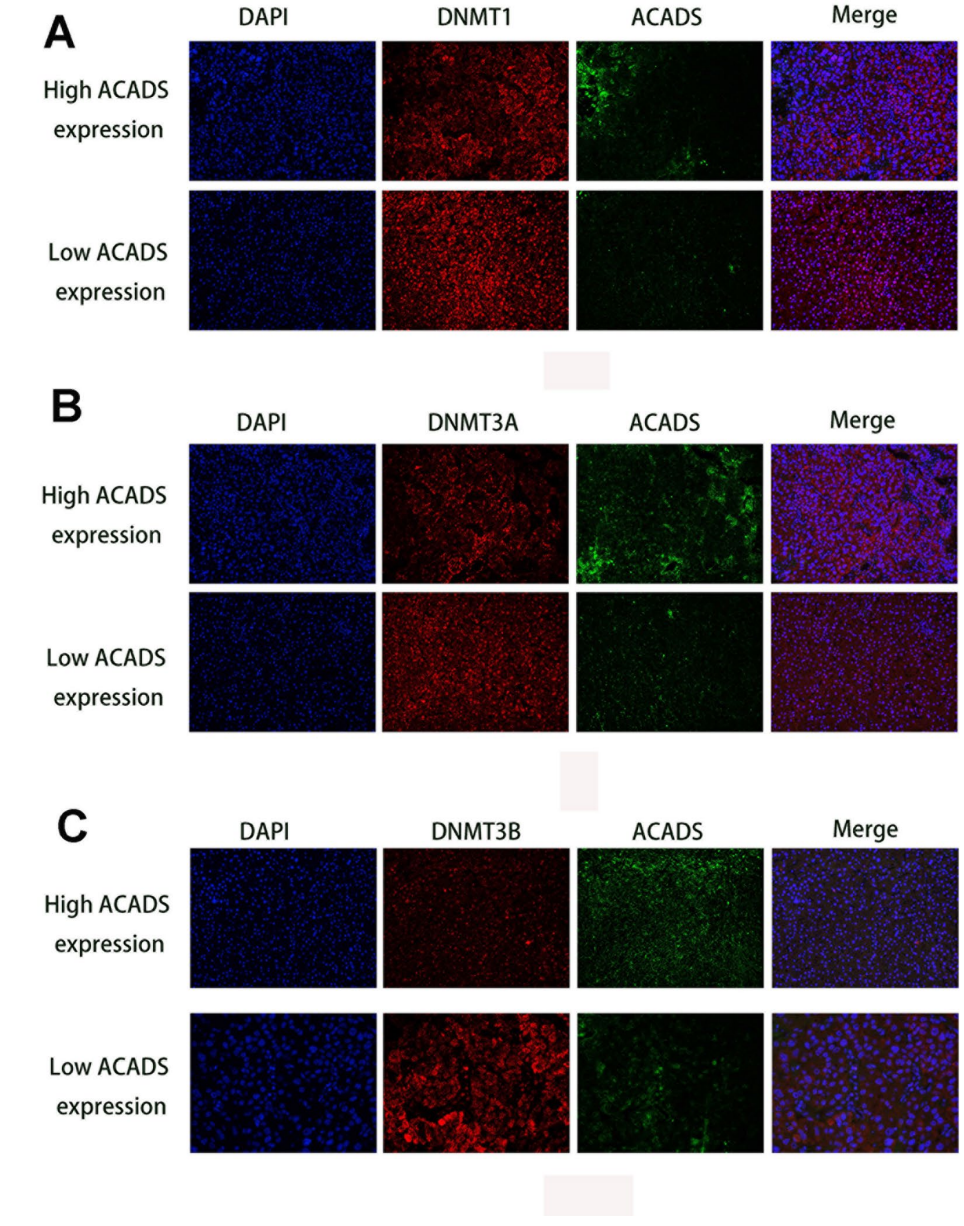
**Immunofluorescence staining results of DNMTs and ACADS in HCC microarrays.** (**A**) Immunofluorescence staining of DNMT1 (red) and ACADS (green) in high and low ACADS groups. (**B**) Immunofluorescence staining of DNMT3A (red) and ACADS (green) in high and low ACADS groups. (**C**) Immunofluorescence staining of DNMT3B (red) and ACADS (green) in high and low ACADS groups.

Recent epigenetic studies have shown that DNA methyltransferase (DNMTs) seems to play an important role in DNA methylation [17, 18]. Therefore, it is useful to check whether or not the methylation level of ACADS is affected by DNMTs. With the help of data found on the Starbase database, it was found that the expression of ACADS is negatively correlated with that of DNMT1, DNMT3A, and DNMT3B (three key types of DNA methyltransferase, Figure 6A). The interactions between ACADS and DNMTs by were examined and studied by the use of immunofluorescence staining of DNMTs and ACADS and HCC microarrays. HCC tissues were characterized and classified into high ACADS expression and low ACADS expression HCC tissues based on their ACADS expression levels and they were grouped accordingly. Compared with the low ACADS expression group, the tissues of the high ACADS expression showed lower DNMTs expression (Figure 7). This result confirmed the hypothesis mentioned above. Thus, it can be concluded that the expression of ACADS might be regulated by DNMTs during the DNA methylation process. In order to further prove the regulatory effects of DNMTs on ACADS, the expression of DNMTs was downregulated by DNMT gene knockdown (which included all three main variants of DNMT - DNMT1, DNMT3A, and DNMT3B). It was observed that, when DNMT function was impeded with the help of a DNA methylase inhibitor (5-Aza), the expression of ACADS rebounded back to normal levels. (Figure 6B–6G, *P<0.05*).

## DISCUSSION

In the current study, gene expression and protein–protein expression analyses using the data contained in public databases were performed in order to identify potential genes that are strongly associated with HCC. By comparing the characteristics of the 282 cancer tissue samples with those of 47 normal tissue samples, it was found that 78 genes were significantly upregulated and 65 genes were significantly downregulated in the HCC samples. Among the 65 downregulated genes, with the help of PPI network analysis, ten “hub genes” were identified and it seems highly likely that these “hub genes” can serve as potential research and therapeutic targets. Earlier studies have demonstrated that some of these genes and their low-expression levels may be associated with HCC. However, ACADS was never reported to play a role in oncogenesis of any kind let alone HCC. Besides, the results of the analytical experiments using the GEPIA database and the Kaplan-Meier survival analysis showed that ACADS was downregulated in HCC and that the ACADS expression level could have prognostic value for HCC patients.

To further explore the function of ACADS in HCC, loss-of-function analyses were employed to understand the biological function of ACADS in HCC cells. In ACADS silenced Huh7 and HCCLM3 cell samples, CCK-8 and colony formation assays confirmed that ACADS could suppress the proliferation of HCC cells. In addition, the cell migration and invasion assays identified that ACADS was associated with HCC cell metastasis. Previous studies revealed that apoptosis plays a vital role in tumor proliferation. So, the mitotracker and hochest3342 combined staining assay was performed to confirm the apoptotic changes in the two cell lines after ACADS siRNA transfection. It was discovered that ACADS could modulate HCC proliferation by influencing apoptosis. Following this observation, *in vivo* experiments were carried out to confirm the results mentioned above. There are still several limitations affecting the effectiveness of the *in vivo* experiments associated with this study and furthermore. the precise biological mechanism by which ACADS influences HCC proliferation, migration, and invasion still needs to be investigated. And in this study, we just used two HCC cell lines to prove our hypothesis, that might be another limit in this research.

Epigenetic modifications, DNA methylation in particular, seems to be associated with the regulation of the magnitude of several diseases [15]. Several aberrantly methylated genes are often shared among different types of cancer cells in general. This is of particular significance in the cancers of the gastrointestinal tract [19, 20]. A recent study has shown that the regulation of DNA methyltransferases (DNMTs) (which are key enzymes that are involved in catalyzing the methylation of different sites of DNA) is closely associated with the expression of various tumor suppressor genes [21]. This observation led us to conduct experiments which focused on DNMT knockdown. The reduced expression of DNMTs owing to the knockdown led to a significant increase in the expression of ACADS in HCCLM3 and Huh7 cells. From the TCGA database, we found that the tumor tissues showed higher methylation levels as compared to their normal liver tissue counterparts at the sites cg01535453, cg08618068, and cg10174836 - which are the target sites of the ACADS CpG island. It can therefore be suggested that DNMTs may play important roles in the regulation of ACADS expression during DNA methylation.

In summary, the experimental and analytical findings and data obtained during the course of this study revealed that ACADS plays an important role in HCC. Our findings not only provide novel insights on the functional characterization of ACADS in HCC, but they also provide a novel methylation biomarker for the diagnosis and prognosis of HCC which can be of great use in future research.

## MATERIALS AND METHODS

### Cell culture

All of the cell lines utilized during the course of this study were obtained from the Cell Bank of Type Culture Collection of the Chinese Academy of Sciences, The Shanghai Institute of Cell Biology and The Chinese Academy of Sciences. All cell lines were maintained in Minimum Essential Media (Cat. No. GNM-41500-S, Genom, China) containing 10% fetal bovine serum (Moregate Biotech, Australia) and the cell cultivation was conducted in a 37°C, 5% CO2 humidified incubator.

### Data sources

The gene expression datasets analyzed in this study were obtained from the GEO database (https://www.ncbi.nlm.nih.gov/geo/). A total of 1,387 series which were associated with human hepatocellular carcinoma were retrieved from the database. After a careful review, specific gene expression profiles namely, GSE87630, GSE89377, and GSE112790 were selected. All of the data utilized in the study is freely available online, and no animal or human experimentation was associated with this study.

### Data processing of DEGs

The GEO2R online analysis tool (https://www.ncbi.nlm.nih.gov/geo/geo2r/) was used to detect the DEGs associated with the control group and the experimental group, and the adjusted P-values and |logFC| values were calculated. Genes that met the cutoff criteria (adjusted *P*<0.05 and |logFC|≥2.0), were considered as DEGs. Statistical analyses were carried out for each dataset, and the intersecting portions was identified using the Venn diagram webtool (bioinformatics.psb.ugent.be/webtools/ Venn/).

### GO and KEGG pathway analysis of DEGs

GO analysis is a common but extremely useful method for large scale functional enrichment research; Genes can be classified into different types, namely, those which are associated with biological processes (BP), those that are associated with molecular functions (MF), and those that are cellular components (CC).

KEGG is a database which collects large amounts of data associated with genomes, biological pathways, diseases, chemical substances, and drugs. The GO annotation analyses and the KEGG pathway enrichment analyses of DEGs involved with this study were performed using the Database for Annotation, Visualization and Integrated Discovery (DAVID) tools (https://david.ncifcrf.gov/). The datasets which met the cutoff criteria (*P*<0.01 and gene counts≥10), were considered statistically significant.

### PPI network construction and hub gene identification

The DEGs were uploaded to the Search Tool for the Retrieval of Interacting Genes (STRING) database analysis platform to obtain a PPI map. The PPI pairs which possessed a combined score>0.4 were then extracted. Subsequently, the PPI network was visualized with the help of the Cytoscape software (http:/www.cytoscape.org/). The Nodes with higher degrees of connectivity tend to be more essential in maintaining the stability of the entire network. The top ten genes (ranked according to their centrality indices) were considered to be potential hub gene candidates.

### RNA isolation and quantitative real-time PCR (qRT-PCR)

Total RNAs were isolated using the TRIzol reagent (TaKaRa, China) and then transcribed into cDNAs with the help of the PrimeScript RT Reagent Kit (TaKaRa, China). An ABI 7500 FAST Real-Time PCR system (Applied Biosystems, USA) was used for the quantitative real-time PCR analysis which also employed the use of a SYBR Green PCR Kit (Takara, China). Relative quantification of mRNA expression was based on the 2ΔΔCt method and the quantification was done after the data was normalized with respect to the endogenous reference GAPDH levels. The primers used are shown in Supplementary Table 1.

### Western blot analysis

The Soluble protein components of the cells were collected after cell lysis. The protein concentration was determined by the Bradford assay (Bio-Rad). Equal amounts of lysate were applied to a 10% NuPAGE Bis-Tris Gel (Invitrogen) and gel electrophoresis was performed. The lysates were then transferred onto PVDF membranes for 90mins, and then the reactive sites were blocked with the help of a 5% skimmed milk solution. The membranes were washed once with TBST, then incubated for overnight at 4°C with relevant primary antibodies (ACADS, 16623-1-AP, proteintech; DNMT1, 24206-1-AP, proteintech; DNMT3A, 19366-1-AP, proteintach; DNMT3B, ab2851, abcam). The membranes were then washed again for three times with TBST after which the secondary antibody (Goat Anti-Rabbit IgG 1:5000) was added and the samples were incubated for a duration of 2 hours at room temperature. Upon the conclusion of this step, the membranes were subjected to three washes with TBST and then suitable amounts of ECL liquid was added and the membranes were placed in a darkroom for the reaction to proceed. A GAPDH solution (1:5000 dilution, Sigma-Aldrich) was used as the loading control reagent.

### RNA silencing

ACADS short interfering (si) RNA (two different sequences tested; si1, GGAGTTGTTTCCCATTGCA; and si2, GCATCACTGAGATCTACGA) and negative control siRNA (UUCUCCGAACGUGUCACGU) were purchased from Origene Technologies, Inc. (Rockville, MD, USA). siRNA (10 nM) was transfected with 5 μl/ml Lipofectamine 2000 (Invitrogen; Thermo Fisher Scientific, Inc., Waltham, MA, USA). Firstly, cells (2×105 per well) were seeded in 6-well plates and incubated at 37°C for 24 h. A total of 5 μl siRNA was added into 250 μl MEM, and 5 μl Lipofectamine 2000 was added to another 250 μl MEM, and the two solutions were incubated at 24°C for 5 min. Next, the two solutions were mixed together and incubated at 24°C for 20 min. The supernatant of the wells was removed, and the transfection solution and 1.5 ml MEM medium were added and incubated at 37°C for 8 h. After 8 h, the supernatant was removed and 2 ml MEMmedium with 10% FBS were added, and the following experiments were performed at 24 h after transfection.

For stable ACADS knockdown, ACADS short hairpin RNA (shRNA) lentiviral vectors (lenti-shRNA/H2A.Z) and negative control lentivirus [expressing green fluorescent protein (GFP)] were purchased from GeneChem Co., Ltd. (Shanghai, China). Target cells were infected with lentivirus of shACADS and negative control groups at the concentration of 1×108 TU/ml. Cells (2×105 per well) were seeded in 6-well plates and incubated for 24 h. Next, the supernatant was removed, and 1 ml RPMI-1640 was mixed with 1×108 TU lentivirus and 1μl polybrene and incubated at 37°C for 6 h. After 6 h, the supernatant was changed with culture medium. The concentration of lentivirus and the conditions of infection were according to the manufacturer’s instructions. Cells expressing shACADS were selected in culture medium containing puromycin (3 μg/ml). The ACADS shRNA target sequence was GGAGTTGTTTCCCATTGCA.

### Proliferation and colony formation assay

The cell proliferation studies were carried out as follows. Initially, HCC cells were taken in 96-well plates at a concentration of 1200 cells/well. Cell viability and the growth characteristics was determined by calculating the absorbance of the samples at a wavelength of 450nm using the Cell-Counting Kit (CCK)-8 (Dojindo). Colony stimulation and formation were studied by introducing cell samples into 6-well plates at a concentration of 2000 cells/well and the cells were cultured by letting them grow at 37°C for 10–16 days keeping in line with the characteristics of each cell line. The cells were then fixed with the help of 100% methanol and stained with 0.1% crystal violet. Macroscopic colonies were counted with the help of Image-Pro Plus 5.0 (Media Cybernetics).

### Cell migration and invasion assays

Millicell Cell Culture Inserts (24-well plate; 8μm pore size; Millipore, USA) were used to perform cell migration and invasion assays. In the case of the cell invasion assays, the inserts were coated with 35 μl of a mixed liquor solution containing Matrigel (BD Biosciences, USA)/MEM at a 1:7 ratio and the samples were incubated in this solution for a duration of 3 hours in a 37°C humidified incubator. For every insert, a sample of 3×10^4^ Huh7 cells or 5×10^4^ HCCLM3 cells in serum-free medium was inoculated into the upper chamber. The culture medium which contained 10% FBS was added into the lower chamber. The cells were incubated at 37°C and 5% CO2. The cell samples were incubated for a duration of 48 hours in the migration assay experiments and for the duration of 72 hours in the invasion assay experiments. The cells on the lower surface of the membrane were stained by using the Wright-Giemsa Stain Kit (Nanjing Jiancheng Bioengineering Institute, China).

### Nude mice xenograft experiment

All the animal experiments associated with this study were conducted in accordance with the rules and regulations provided by the National Institutes of Health (Guide for the Care and Use of Laboratory Animals, 2011). The cell samples were resuspended in 100 μl PBS and then injected subcutaneously into the left flank of the mice (5×10^6^ cells per mouse for HCCLM3). The tumor volume was calculated according to the following formula: TV (tumor volume) = [(larger diameter) × (smaller diameter)^2^]/2. The subcutaneous tumors were removed and weighed on the 30^th^ day. Each group consisted of at least 6 mice.

### Statistical analysis

All the Statistical tests and analyses performed to measure and quantify the relevant parameters associated with the current study were performed using the SPSS 17.0 software (SPSS). The quantitative data from the control and the experimental groups were compared by using the Student t test or the Wilcoxon signed-rank test. The chi-square test and the Fisher’s exact test were used to evaluate any potential association between ACADS expression and the clinicopathological parameters relevant to the study. The Statistical significance level was set at *P < 0.05, **P< 0.001.

## Supplementary Material

Supplementary Figures

Supplementary Table
