# Supplementary material for: ACADS acts as a potential methylation biomarker associated with the proliferation and metastasis of hepatocellular carcinomas
Source: Aging (Albany NY). 2019 Oct 25;11(20):8825–44. doi: 10.18632/aging.102292 (PMC6834414; doi:10.18632/aging.102292)
Supplement: Supplementary Figures [file aging-11-102292-s002.pdf]

SUPPLEMENTARY FIGURES

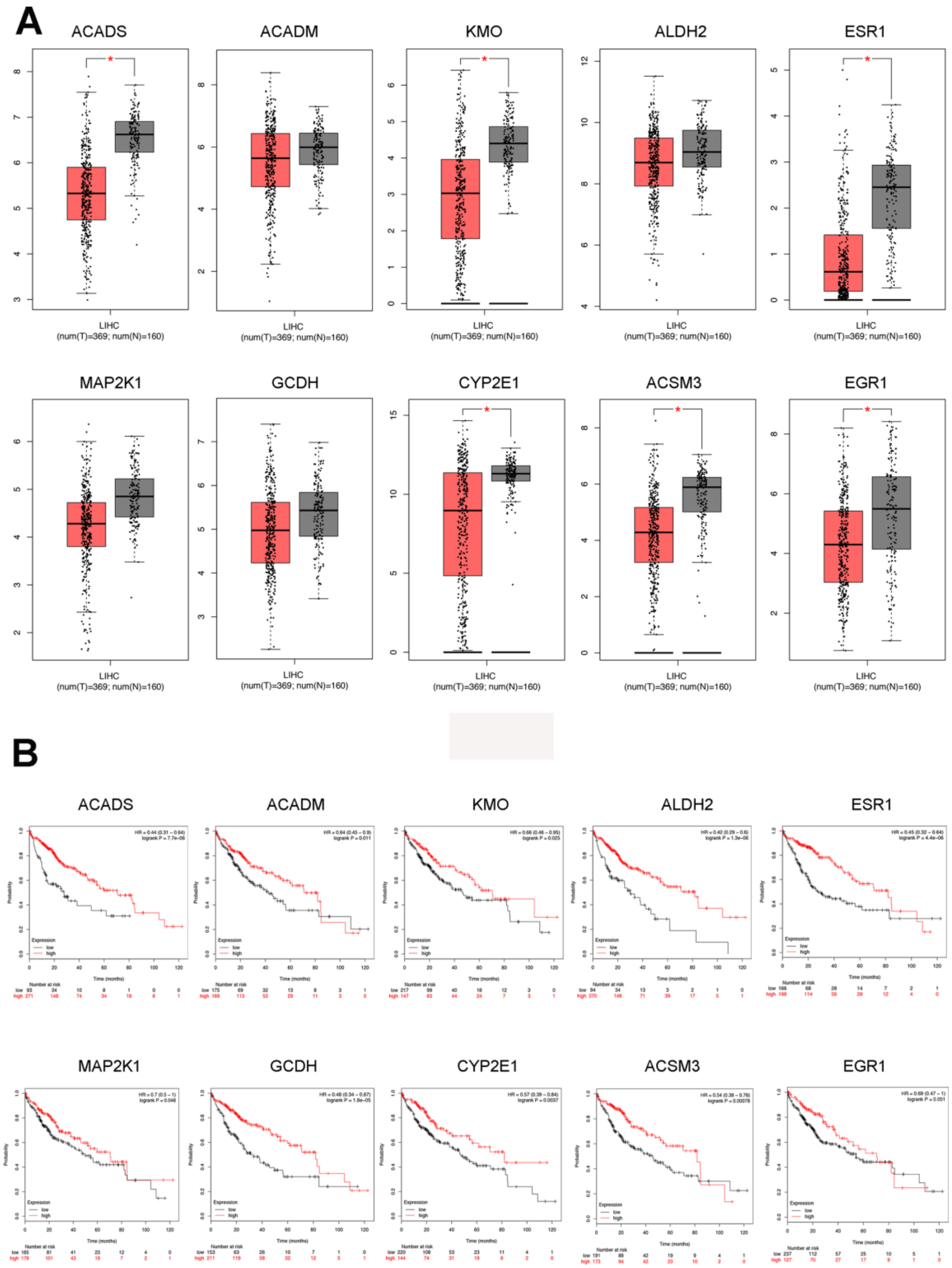

Supplementary Figure 1. (**A**) These figures depict the gene expression profiles of the ten downregulated hub genes in normal tissues and HCC tissues and these were obtained with the assistance of the GEPIA database. (**B**) These figures depict the relationship between overall survival and gene expression in the ten hub downregulated hub genes in HCC tissues and their normal counterparts. (* *P*<0.05, ***P*<0.01).

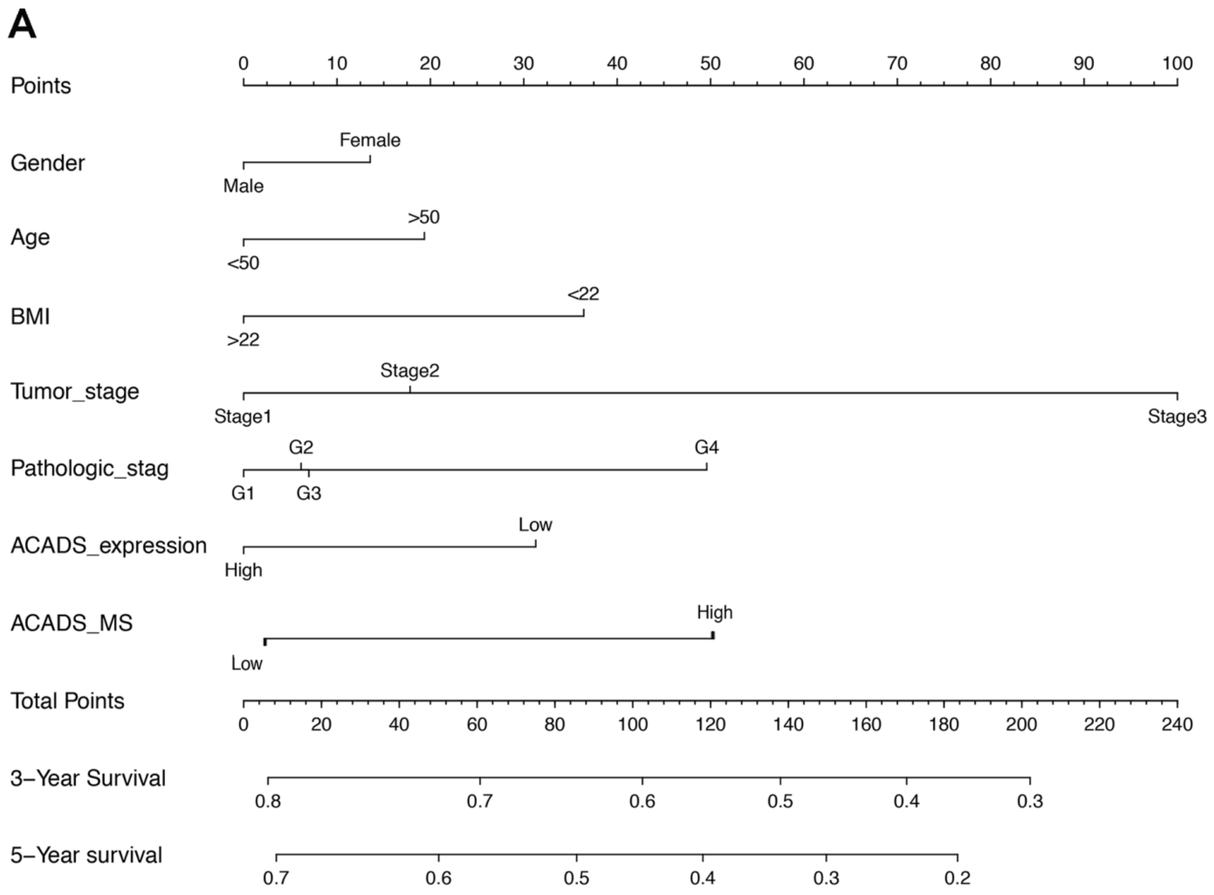

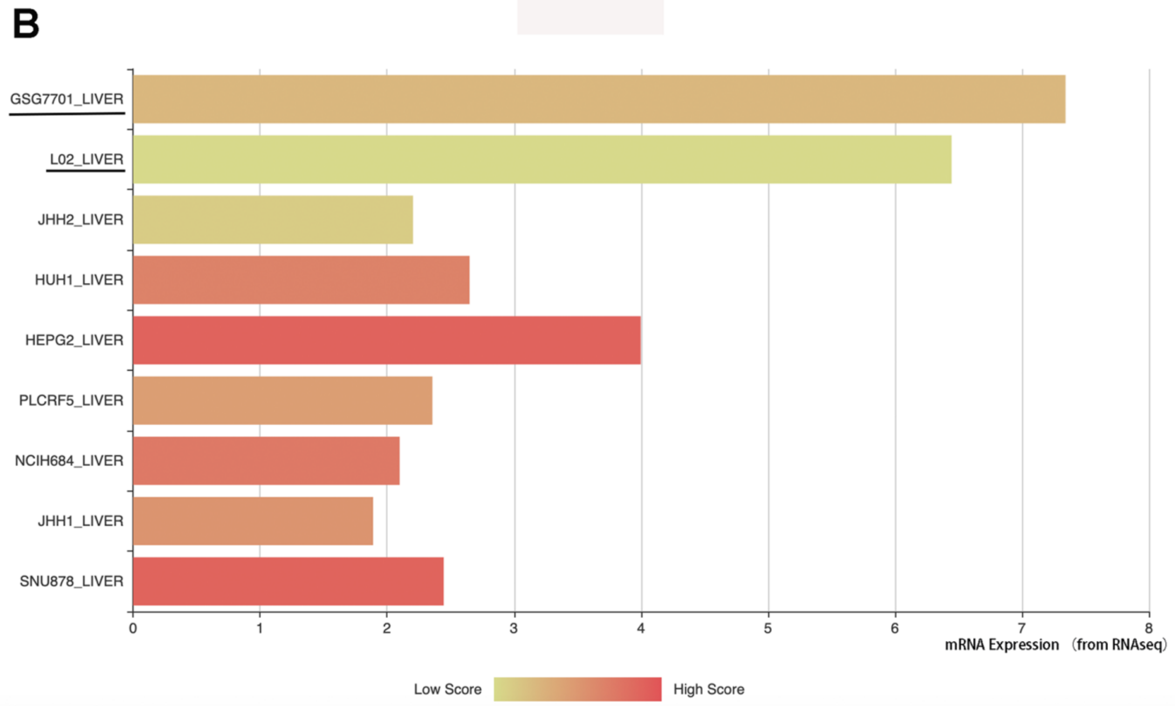

Supplementary Figure 2. (**A**) Using cell line webtool, we checked the ACADS expression in 2 normal liver cells and 7 HCC cell lines. (**B**) The survival-model established based on ACADS expression and methylation level in HCC patients.

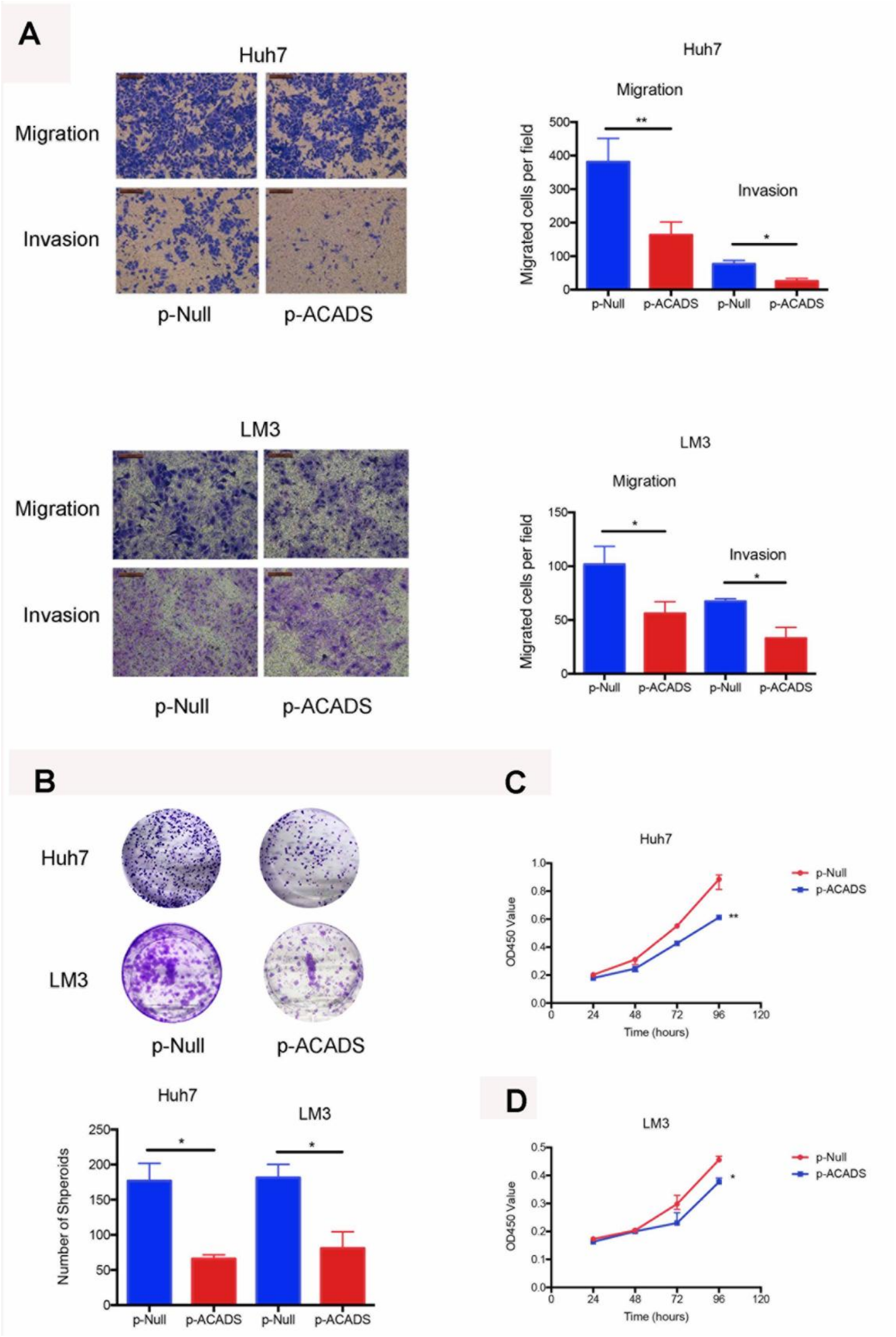

Supplementary Figure 3. (**A**) The overexpression of ACADS reduced both the migration and invasion potentials in Huh7 and HCCLM3 cells. Representative images are shown at the bottom. (**B**–**D**) CCK-8 assays and colony assays showed that the proliferation of HCC cells was reduced following the overexpression of ACADS. (* P<0.05, **P<0.01).

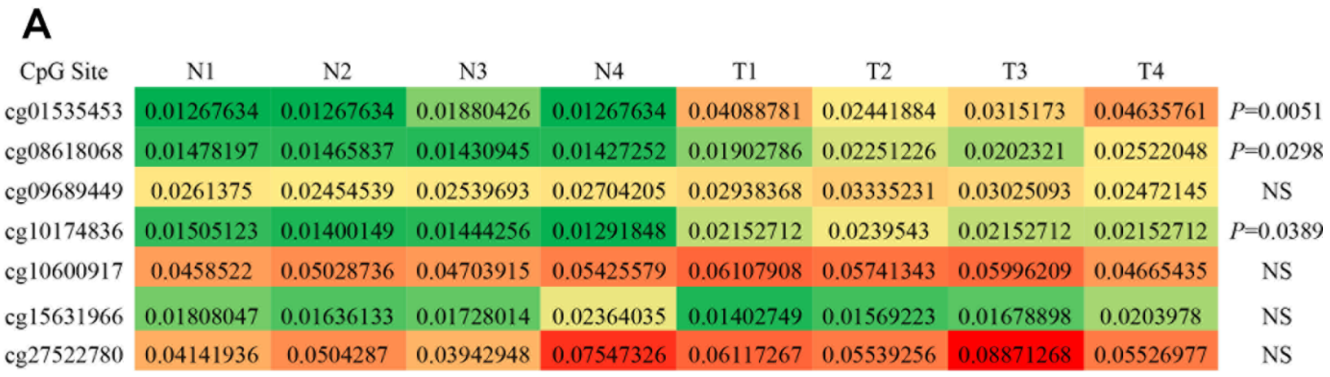

A

CpG Site	N1	N2	N3	N4	T1	T2	T3	T4	
cg01535453	0.01267634	0.01267634	0.01880426	0.01267634	0.04088781	0.02441884	0.0315173	0.04635761	*P*=0.0051
cg08618068	0.01478197	0.01465837	0.01430945	0.01427252	0.01902786	0.02251226	0.0202321	0.02522048	*P*=0.0298
cg09689449	0.0261375	0.02454539	0.02539693	0.02704205	0.02938368	0.03335231	0.03025093	0.02472145	NS
cg10174836	0.01505123	0.01400149	0.01444256	0.01291848	0.02152712	0.0239543	0.02152712	0.02152712	*P*=0.0389
cg10600917	0.0458522	0.05028736	0.04703915	0.05425579	0.06107908	0.05741343	0.05996209	0.04665435	NS
cg15631966	0.01808047	0.01636133	0.01728014	0.02364035	0.01402749	0.01569223	0.01678898	0.0203978	NS
cg27522780	0.04141936	0.0504287	0.03942948	0.07547326	0.06117267	0.05539256	0.08871268	0.05526977	NS

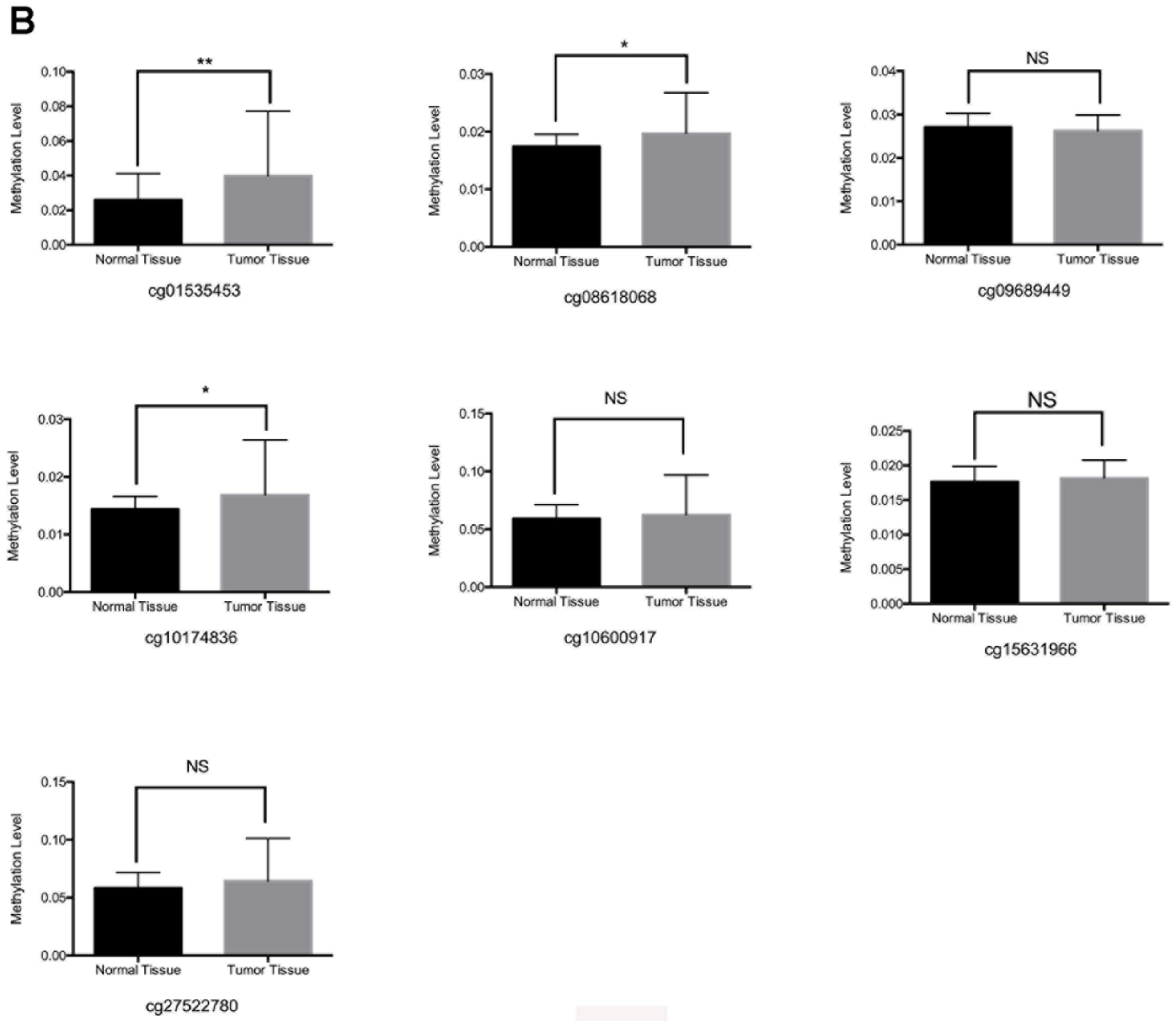

Supplementary Figure 4. It can be noted that tumor tissues exhibited higher methylation levels as compared to the normal liver tissues at the sites cg01535453, cg08618068, and cg10174836 both in the samples sequencing data (**A**) from our center and TCGA darabase (**B**) – which are the target sites of the ACADS CpG islands.
