# Supplementary material for: ACADS acts as a potential methylation biomarker associated with the proliferation and metastasis of hepatocellular carcinomas
Source: Aging (Albany NY). 2019 Oct 25;11(20):8825–44. doi: 10.18632/aging.102292 (PMC6834414; doi:10.18632/aging.102292)
Supplement: Supplementary Table [file aging-11-102292-s001.pdf]

SUPPLEMENTARY TABLE

Supplementary Table 1. Primer sequences of 4 genes.

Gene Symbol	Item	Sequence (5′ -> 3′)
ACADS	Forward Primer	CGGCAGTTACACACCATCTAC
	Reverse Primer	GCAATGGGAAACAACTCCTTCTC
DNMT1	Forward Primer	AGAACGGTGCTCATGCTTACA
	Reverse Primer	CTCTACGGGCTTCACTTCTTG
DNMT3A	Forward Primer	CCGATGCTGGGGACAAGAAT
	Reverse Primer	CCCGTCATCCACCAAGACAC
DNMT3B	Forward Primer	AGGGAAGACTCGATCCTCGTC
	Reverse Primer	GTGTGTAGCTTAGCAGACTGG
